# Sex-specific Association Between Uric Acid and Outcomes After Acute Ischemic Stroke: A Prospective Study from CATIS Trial

**DOI:** 10.1038/srep38351

**Published:** 2016-11-30

**Authors:** Li-Hua Chen, Chongke Zhong, Tan Xu, Tian Xu, Yanbo Peng, Aili Wang, Jinchao Wang, Hao Peng, Qunwei Li, Zhong Ju, Deqin Geng, Jintao Zhang, Yongqiu Li, Yonghong Zhang, Jiang He

**Affiliations:** 1 Department of Epidemiology, School of Public Health and Jiangsu Key Laboratory of Preventive and Translational Medicine for Geriatric Diseases, Medical College of Soochow University, Suzhou, China; 2 Department of Epidemiology, Tulane University School of Public Health and Tropical Medicine, New Orleans, LA, USA; 3 Department of Neurology, Affiliated Hospital of Nantong University, Nantong, Jiangsu, China; 4 Department of Neurology, Affiliated Hospital of North China University of Science and Technology, Hebei, China; 5 Department of Neurology, Yutian County Hospital, Hebei, China; 6 Department of Epidemiology, School of Public Health, Taishan Medical College, Shandong, China; 7 Department of Neurology, Kerqin District First People’s Hospital of Tongliao City, Inner Mongolia, China; 8 Department of Neurology, Affiliated Hospital of Xuzhou Medical College, Jiangsu, China; 9 Department of Neurology, the 88th Hospital of PLA, Shandong, China; 10 Department of Neurology, Tangshan Worker’s Hospital, Hebei, China

## Abstract

The relationship between serum uric acid (UA) and outcomes after acute ischemic stroke remains debatable in human studies, and the sex effect on this association has yet to be explored. Here, we investigated these associations in a prospective study from the China Antihypertensive Trial in Acute Ischemic Stroke. Baseline UA levels were measured in 3284 acute ischemic stroke patients. Primary outcome was defined as a combination of death and major disability (modified Rankin Scale score ≥3) at 3 months. UA levels were significantly higher in men than women (310.6 ± 96.1 vs 257.5 ± 89.9 μmol/L, *P* < 0.001). The association between serum UA and the primary outcome was appreciably modified by sex (*P*-interaction = 0.007). After multivariate adjustment, a high serum UA was associated with a decreased risk of primary outcome in men [odds ratio (OR), 0.63; 95% confidence interval (CI), 0.44–0.91; *P*-trend = 0.01] but not in women (OR, 1.29; 95% CI, 0.83–2.01; *P*-trend = 0.15), when two extreme quartiles were compared. Subgroup and sensitivity analyses further confirmed these sex-specific findings. Our study indicated that there was a sex-specific association between serum UA and prognosis of acute ischemic stroke. Elevated serum UA was positively associated with better prognosis in men, but not in women.

Stroke-related morbidity and mortality are one of the main public health concerns. Although treatment options for the acute ischemic stroke have recently made progress, the condition of many patients does not improve significantly due to various reasons (for example, missed thrombolytic therapy time window). As a result, stroke remained a main cause of long-term disability and mortality^1^,^2^,^3^. Oxidative stress is a major contributor to brain damage in patients with ischemic stroke^4^. Blocking the activation of free radicals and preventing the death of nerve cells in the ischemic penumbra is considered to be a potential effective approach to ameliorate the long-term outcomes of stroke^3^,^5^.

Uric acid (UA) is the metabolic end product of purine metabolism in humans, and the most abundant aqueous antioxidant^6^,^7^. It has been proposed to be a neuroprotective agent via its capacity to prevent oxidative stress caused by reactive nitrogen and oxygen species, thus UA could be an important determinant of stroke susceptibility^8^,^9^. However, the results of epidemiological studies investigating the effect of serum UA levels on the prognosis of acute ischemic stroke are conflicting because of previous studies being limited to small single-center series or poor study design. Some studies suggested serum UA was protective for the prognosis of acute ischemic stroke^10^,^11^,^12^,^13^, whereas others indicated that it had no such effect^14^,^15^ or even increased the risk of poor outcome and future vascular events after acute stroke^16^,^17^. It is well known that UA levels are higher in men, and a sex-difference in the relationship between serum UA and risk of cardiovascular disease had been reported^18^,^19^,^20^. So there may be a sex-specific association between UA and prognosis of acute ischemic stroke. Until now, large sample size and well-designed studies about the relationship between UA and prognosis of acute ischemic stroke are rare, and evidences in statistically strong power are urgently needed.

Herein, we sought to investigate the association between admission serum UA levels and 3-month outcomes after acute ischemic stroke in both men and women using data from the China Antihypertensive Trial in Acute Ischemic Stroke (CATIS).

## Results

### Baseline Characteristics

As expected, men had higher level of serum uric acid than women (310.6 ± 96.1 μmol/L vs 257.5 ± 89.9 μmol/L, *P* < 0.001) (Supplementary Table 1). Among men, participants with higher serum UA tended to be younger, had higher DBP, BMI, TG, TC, LDL-C, white blood cell, creatinine, prevalence of history of hypertension, current use of antihypertensive medications, history of hyperlipidemia, history of coronary heart disease, history of chronic kidney disease and thrombotic ischemic stroke, and had lower time from onset to randomization, HDL-C, blood glucose, baseline NIHSS and mRS score, and prevalence of history of diabetes mellitus than those with lower serum UA; among women, compared with those with lower UA levels, age, BMI, TC, TG, LDL-C, creatinine, and prevalence of history of hypertension, current use of antihypertensive medications, history of CHD and embolic ischemic stroke were higher, while time from onset to randomization and HDL-C were lower in participants with higher serum UA levels (Table 1).

### Association Between Serum UA and Prognosis

After 3-month follow-up, a total of 484 (23.2%) men and 323 (27.0%) women patients with poor outcome were documented (Table 2). The association between serum UA and the primary composite outcome was appreciably modified by sex (*P*-interaction = 0.007). After adjustment for age, current smoking, alcohol consumption, history of hypertension, baseline NIHSS score and other potential covariates, the ORs for the highest vs lowest quartiles of serum UA were 0.63 (95% CI, 0.44–0.91; *P*-trend = 0.01) for men and 1.29 (95% CI, 0.83–2.01; *P*-trend = 0.15) for women. Each SD increase in UA was associated with 14% (95% CI: 2–24%) decreased risk of primary outcome for men, but not for women (Table 3). In subgroup analyses stratified by stroke onset age, baseline SBP, current smoking, alcohol consumption and receiving immediate BP reduction, the modest inverse associations between serum UA and primary outcome were observed in all subgroups, and reached significant in several subgroups among men; however, no significantly subgroup was evidenced in women (Table 4). Moreover, no significant interaction between serum UA with these interested factors, in relation to primary outcome was observed among men and women patients (*P*-interaction >0.05 for all).

The sensitivity analyses further strengthened the findings that elevated serum UA was associated with lower risk of poor outcome in men but not in women (*P*-interaction <0.008 for all), when randomized treatment or baseline serum creatinine were adjusted in multivariable models or patients with chronic kidney disease were excluded (Supplementary Table 2).

## Discussion

In this large prospective multicenter study from the China Antihypertensive Trial in Acute Ischemic Stroke, we observed a dose-response association between higher serum UA and a better prognosis of acute ischemic stroke in men, but not in women, even after adjustment for potential confounders of this relationship. Furthermore, the modest inverse associations between serum UA and primary outcome were observed in all subgroups among men, but not among women. Thus, the present study strongly supported higher serum UA was an independent predictor of better prognosis of acute ischemic stroke in men.

Our finding that uric acid is protective for prognosis of acute ischemic stroke in men may be explained by several possible biological mechanisms. As it is known that excessive extracellular accumulation of glutamate is a major factor contributing to neuron death in the ischemic penumbra zone^21^. Glutamate activates its receptor N-methyl-D-aspartate and further leads to cytoplasmic accumulation of Ca^2+^. The accumulated Ca^2+^ activates Ca^2+^-dependent enzymes, including calpains and caspases, and finally leads to apoptotic cell death^21^. Previous study showed that uric acid protected rat hippocampal neurons against glutamate-induced cell death^22^. Uric acid was also very effective in clearing superoxide and in detoxifying peroxynitrite, suggesting an important role in suppressing membrane lipid peroxidation^23^. Low total peroxyl radical trapping potential of plasma was associated with greater lesion volumes and neurological impairment in patients with stroke^24^.

The neuroprotective role of uric acid has also been explored among population studies^10^,^11^,^12^,^13^. Study conducted by Wu *et al*. showed that lower uric acid levels were correlated with poor function outcomes after acute ischemic stroke^12^. Chamorro *et al*. found that in patients with acute ischemic stroke, there was a 12% increase in the odds of good clinical outcome for each milligram per deciliter increase of serum uric acid^4^. Similarly, Amaro *et al*.’ prospective study suggested increased uric acid levels are associated with better outcome in stroke patients treated with reperfusion therapies^11^. Moreover, a recent systematic review and meta-analysis of 10 studies found that compared with low serum uric acid level, high serum uric acid level was associated better outcome after acute ischemic stroke^13^.

The current study supported the neuroprotective role of uric acid, and more importantly, we observed a clear sex difference in the association of uric acid levels with 3-month outcomes after acute ischemic stroke. The dose-response association between higher serum UA and a better prognosis of acute ischemic stroke was only found in men. Similar sex difference pattern has been consistently observed in a study including 218 patients treated with intravenous thrombolysis^25^. They found increased serum UA levels might be associated with better outcomes of acute ischemic stroke particularly in men but not in women. A sex-difference protective effect of UA on nervous system disease (such as Parkinson disease) had also been reported^26^,^27^. For example, a recent nested case-control study based on 3 ongoing US cohorts suggested that men, but not women, with higher urate concentrations had a lower future risk of developing Parkinson disease^26^. The biological mechanisms underlying such sex specificity remain unclear. As we know, there is a sex difference in UA level, women usually have a lower UA level than men. Several previous studies have demonstrated that higher UA levels were significantly related with the development of hypertension and metabolic syndrome in women than those in men^20^,^28^,^29^. This could play a part in the observed sex difference in the relationship between UA and acute ischemic stroke because higher uric acid concentrations are associated with these stroke risk factors, which in turn might offset the potential neuroprotective effects of uric acid. Interestingly, in the recent URICO-ICTUS trial to test the efficacy of combined treatment with 1000 mg uric acid and rt-PA, they found that UA therapy doubled the effect of placebo to attain an excellent outcome in women, but not in men^30^. Women acquired greater clinical benefits after UA supplement than men, this may be partly because the basal levels of UA are lower in women than that in men, and women more need additional antioxidant to scavenge free radicals, to prevent free radicals-induced neuron damage, and then benefit more from this UA intervention^23^. In our present study, we did not find dose-response association between serum UA and prognosis of acute ischemic stroke in women. We cannot eliminate the possibility that sample size of women may contribute to this sex-specific finding. Our study included 1195 women which were about 50% of men (2089) included; relatively small sample might decrease the statistical power for examining the association between UA levels and prognosis of acute ischemic stroke in women. We recruited more men in our study because the incidence rate for acute ischemic stroke of men in China is much higher than women; and the ratio of men to women and the baseline characteristics of participants in this study were very similar to those from the China National Stroke Registry^31^. Further studies with large sample size are required to verify our findings and clarify the mechanisms of sex-specific relationship between serum UA and prognosis of acute ischemic stroke.

Several characteristics of our study deserve mention. The current study represents the largest longitudinal prospective study investigating the independent association between serum UA and prognosis of acute ischemic stroke, and a relatively large sample size enabled us to perform several subgroup and sensitivity analyses to confirm our sex-specific findings. All data were collected with rigid quality control. Compared with previous studies, we collected several important variables which could confound the observed association, including serum creatinine levels, current use of antihypertensive medications, family history of stroke and severity of stroke. Furthermore, uric acid was measured on admission, before its concentration could be confounded by the intensity of fluid replacement. Thus, serum UA levels might serve as an important predictor of stroke prognosis in men with acute ischemic stroke. The limitation of our study was that the findings were based on a single UA measurement but not on serial measurements, therefore, our data set was not adequate to evaluate the serum UA time response as a function of stroke severity. Another limitation was that the information about antioxidant use was not collected in this study, which might influence the association between serum UA and prognosis of acute ischemic stroke. Moreover, this study is a post hoc analysis of a randomized clinical trial, the trial excluded patients with blood pressure of 220/120 mm Hg or greater, or severe diseases, or treated with intravenous thrombolytic therapy, therefore a selection bias may unavoidably be present, and the primary results of the present study may not be generalized to all ischemic stroke patients. However, our subgroup analyses showed that the inverse association between serum UA and prognosis of acute ischemic stroke among men but not women was not modified by receiving immediate BP reduction or not (*P*-interaction >0.05 for both).

In conclusion, our study suggests that men with higher serum uric acid concentrations prone to be with better prognosis of acute ischemic stroke than women. Sex-specific analyses should be given priority in future studies.

## Methods

### Study design and participants

Participants in the current analysis were from the CATIS trial (clinicaltrials.gov Identifier: NCT01840072), a multicenter, single-blind, blinded end-points randomized clinical trial conducted in 26 hospitals across China to test whether employing antihypertensive treatment within the first 48 hours after the onset of an acute ischemic stroke would reduce death and major disability at 14 days or hospital discharge^32^. Briefly, a total of 4071 patients 22 years or older who had ischemic stroke, confirmed by computed tomography (CT) or magnetic resonance imaging (MRI) of the brain within 48 hours of symptom onset and who had an elevated systolic blood pressure between 140 mm Hg and less than 220 mm Hg were recruited in this trial. Patients with a systolic blood pressure (SBP) ≥220 or diastolic blood pressure (DBP) ≥120 mmHg, severe heart failure, acute myocardial infarction or unstable angina, atrial fibrillation, aortic dissection, cerebrovascular stenosis, or resistant hypertension; those in a deep coma; and those treated with intravenous thrombolytic therapy were excluded. 716 patients were excluded because they refused to offer blood samples, or some collected samples were hemolyzed in storage or transport, or failed to measure serum UA levels. There were no remarkable differences between enrolled and excluded patients in baseline characteristics, such as age (enrolled, 62.6 ± 11.0 years vs excluded, 62.2 ± 10.5 years; *P* = 0.490) and sex (men, enrolled 63.7% vs excluded 65.5%; *P* = 0.345). After 3-month follow-up, 71 patients were lost, the follow-up rate was 97.9%. Finally, a total of 3284 acute ischemic patients were included in this study.

This study was approved by the institutional review boards at Tulane University in the United States and Soochow University in China, as well as ethical committees at the 26 participating hospitals. Written consent was obtained from all study participants or their immediate family members. All methods were carried out in accordance with the approved guidelines.

### Assessment of serum uric acid concentrations and potential confounders

Blood samples were collected with at least 8 h of fasting within 24 hours of hospital admission. Serum UA concentration was tested by standard laboratory procedures with urate oxidase reagent with an inter-assay coefficient of variation 3 to 5%. Laboratory technicians who performed these measurements were blinded to the clinical characteristics of the study participants.

Data on demographic characteristics, lifestyle risk factors, medical history, clinical laboratory tests, imaging data (CT and MRI) were collected at the time of enrollment. Stroke severity was assessed using the National Institutes of Health Stroke Scale (NIHSS) by trained neurologists at baseline. Three blood pressure measurements were obtained at baseline by trained nurses according to a common protocol adapted from procedures recommended by the American Heart Association^33^. Blood pressure was measured with the participant in a supine position using a standard mercury sphygmomanometer and 1 of 4 cuff sizes (pediatric, regular adult, large adult, or thigh) based on participant arm circumference. Routine laboratory determinations (plasma glucose, blood lipids, etc.) were performed for all enrolled patients in each participating hospital at admission. Low-density lipoprotein cholesterol (LDL-C) concentration was calculated by the use of the Friedewald equation for participants who had less than 400 mg/dL triglycerides. The criterion of dyslipidemia was as follows: total cholesterol ≥6.22 mmol/l or triglyceride ≥2.26 mmol/l or LDL cholesterol ≥4.14 mmol/l or HDL cholesterol <1.04 mmol/l^34^.

### Assessment of outcomes

Participants were followed up in person at 3 months after hospitalization by trained neurologists and research nurses unaware of treatment assignment. The primary outcome was a combination of death and major disability (modified Rankin Scale score ≥3) within 3 months. Secondary outcomes were separately those of death and major disability (modified Rankin scale score of 6 and 3–5, respectively), and an ordinal analysis of the range of scores of the mRS score. Death certificates were obtained for deceased patients. A trial-wide outcomes assessment committee, blinded to treatment assignment, reviewed and adjudicated subsequent outcomes.

### Statistical analysis

Due to a sex-difference in serum UA level was also observed in our participants, therefore, sex-specific results were reported in the current study. Study participants were classified according to sex-specific quartiles of serum UA. Tests for linear trend were performed using covariance analysis for continuous variables, and Chi-square trend analysis for categorical variables. Multivariate nonconditional logistic regression analyses were performed to assess the association between UA and 3-month prognosis. Odds ratios (ORs) and 95% confidence intervals (95% CIs) were calculated for each group with the lowest quartile as the reference. The potential covariates such as age, time from onset to hospitalization, current smoking, alcohol consumption, glucose, SBP, WBC, dyslipidemia, history of hypertension, history of coronary heart disease, history of diabetes mellitus, family history of stroke, current use of antihypertensive medications, and baseline NIHSS score were included in the multivariate model. Interaction between uric acid and sex, in relation to the acute ischemic stroke prognosis, was tested by the likelihood ratio test of models with interaction terms.

We also performed subgroup analyses in men and women, stratified by stroke onset age, baseline SBP, current smoking, alcohol consumption, and receiving immediate BP reduction in multivariate adjusted logistic regression models. Furthermore, several sensitivity analyses were conducted to test the robustness of the findings. Randomized treatment was first included in the multivariable models to adjust the effect of immediate BP reduction during hospitalization. Because serum creatinine levels reflect renal function and may influence the uric acid levels, we then further adjusted serum creatinine; we performed another sensitivity analysis by excluding individuals with chronic kidney disease. All P values were 2-tailed, and a significance level of 0.05 was used. Statistical analysis was conducted using SAS statistical software (version 9.2, Cary, North Carolina, USA).

## Additional Information

**How to cite this article**: Chen, L.-H. *et al*. Sex-specific Association Between Uric Acid and Outcomes After Acute Ischemic Stroke: A Prospective Study from CATIS Trial. *Sci. Rep.* **6**, 38351; doi: 10.1038/srep38351 (2016).

**Publisher’s note:** Springer Nature remains neutral with regard to jurisdictional claims in published maps and institutional affiliations.

## Supplementary Material

Supplementary Tables

## Figures and Tables

**Table 1:** Characteristics of the study participants according to serum uric acid quartiles by sex.

Characteristics*	Men					Women				
	Q1	Q2	Q3	Q4	P_trend_	Q1	Q2	Q3	Q4	P_trend_
Number of subjects	522	521	521	525		298	298	300	299	
Age, y	62.9 ± 10.2	61.3 ± 10.7	60.8 ± 11.4	60.2 ± 11.9	<0.01	64.0 ± 10.7	64.0 ± 10.5	64.1 ± 10.8	66.4 ± 10.1	0.01
Time from onset to randomization, h	16.4 ± 13.3	14.7 ± 12.2	14.9 ± 13.3	14.5 ± 13.4	0.04	17.0 ± 14.2	17.0 ± 13.8	13.5 ± 11.7	15.0 ± 12.7	<0.01
SBP, mm Hg	166.0 ± 16.2	166.9 ± 17.6	164.4 ± 16.0	165.5 ± 17.1	0.21	168.0 ± 17.5	167.2 ± 17.1	166.3 ± 15.6	167.2 ± 18.3	0.43
DBP, mm Hg	96.5 ± 11.3	97.2 ± 11.1	97.4 ± 11.0	98.4 ± 11.7	0.01	95.3 ± 9.4	95.7 ± 10.5	94.8 ± 11.0	94.9 ± 11.6	0.42
BMI, kg/m^2^	24.4 ± 2.6	24.8 ± 2.8	24.9 ± 2.6	25.4 ± 2.8	<0.01	24.6 ± 3.6	25.0 ± 3.2	25.3 ± 3.0	25.7 ± 3.3	<0.01
TG, mmol/L	1.2 (0.9–1.8)	1.4 (1.0–2.0)	1.5 (1.0–2.1)	1.7 (1.2–2.4)	<0.01	1.3 (0.9–1.9)	1.5 (1.1–2.2)	1.7 (1.2–2.4)	1.8 (1.2–2.5)	<0.01
TC, mmol/L	4.7 (4.1–5.4)	4.8 (4.1–5.5)	4.8 (4.2–5.6)	5.0 (4.3–5.6)	<0.01	5.2 (4.5–5.8)	5.2 (4.5–5.9)	5.4 (4.7–6.1)	5.5 (4.7–6.3)	<0.01
LDL-C, mmol/L	2.7 (2.1–3.3)	2.7 (2.2–3.4)	2.8 (2.2–3.5)	2.9 (2.3–3.4)	<0.01	3.0 (2.4–3.6)	3.0 (2.4–3.6)	3.1 (2.6–3.6)	3.1 (2.5–3.8)	0.01
HDL-C, mmol/L	1.2 (1.0–1.5)	1.2 (1.0–1.5)	1.2 (1.0–1.4)	1.1 (1.0–1.4)	<0.01	1.4 (1.1–1.6)	1.3 (1.1–1.6)	1.3 (1.1–1.5)	1.2 (1.1–1.5)	<0.01
GLU, mmol/L	5.9 (5.1–7.6)	5.8 (5.0–7.1)	5.6 (5.0–6.6)	5.7 (5.0–6.7)	<0.01	6.1 (5.3–8.1)	5.8 (5.1–7.5)	5.8 (5.1–7.4)	6.0 (5.1–7.6)	0.20
WBC, 10^9^/L	7.2 ± 2.4	7.3 ± 2.4	7.2 ± 2.4	10.7 ± 53.0	0.05	6.9 ± 2.4	7.0 ± 2.4	7.3 ± 4.2	9.9 ± 41.7	0.08
Creatinine, μmol/L	70.0 ± 19.6	75.4 ± 29.3	78.0 ± 20.0	85.6 ± 28.4	<0.01	57.3 ± 16.3	58.8 ± 17.5	60.2 ± 17.1	70.4 ± 38.0	<0.01
History of hypertension	382 (73.2)	394 (75.6)	416 (79.9)	443 (84.4)	<0.01	233 (78.2)	237 (79.5)	244 (81.3)	263 (88.0)	<0.01
Current use of antihypertensive medications	225 (43.1)	227 (43.6)	241 (46.3)	281 (53.5)	<0.01	162 (54.4)	149 (50.0)	157 (52.3)	192 (64.2)	0.01
History of hyperlipidemia	22 (4.2)	32 (6.1)	36 (6.9)	45 (8.6)	<0.01	15 (5.0)	21 (7.1)	25 (8.3)	26 (8.7)	0.07
History of diabetes mellitus	104 (19.9)	100 (19.2)	77 (14.8)	70 (13.3)	0.01	69 (23.2)	57 (19.1)	48 (16.0)	69 (23.1)	0.75
History of CHD	38 (7.3)	54 (10.4)	58 (11.1)	59 (11.2)	0.03	36 (12.1)	42 (14.1)	39 (13.0)	55 (18.4)	0.05
History of chronic kidney disease	3 (0.6)	3 (0.6)	4 (0.8)	11 (2.1)	0.01	1 (0.3)	3 (1.0)	4 (1.3)	5 (1.7)	0.11
Family history of stroke	91 (17.4)	106 (20.4)	113 (21.7)	106 (20.2)	0.22	56 (18.8)	61 (20.5)	65 (21.7)	58 (19.4)	0.77
Current cigarette smoking	280 (53.6)	280 (53.7)	281 (53.9)	296 (56.4)	0.39	23 (7.7)	30 (10.1)	36 (12.0)	28 (9.4)	0.37
Current alcohol drinking	230 (44.1)	247 (47.4)	254 (48.8)	257 (49.0)	0.10	6 (2.0)	7 (2.4)	15 (5.0)	9 (3.0)	0.21
Ischemic stroke subtype										
Thrombotic	433 (83.0)	414 (79.5)	399 (76.6)	409 (77.9)	0.02	243 (81.5)	231 (77.5)	237 (79.0)	227 (75.9)	0.15
Embolic	14 (2.7)	13 (2.5)	14 (2.7)	19 (3.6)	0.36	6 (2.0)	4 (1.3)	17 (5.7)	15 (5.0)	0.01
Lacunar	93 (17.8)	104 (20.0)	116 (22.3)	108 (20.6)	0.18	59 (19.8)	72 (24.2)	57 (19.0)	64 (21.4)	0.97
Baseline NIHSS score	5 (3–8)	4 (2–7)	4 (2–7)	4 (2–7)	<0.01	5 (3–8)	4 (3–7)	4 (2–7)	4 (3–8)	0.14
Baseline mRS score†	3 (2–4)	2 (2–4)	2 (1–4)	2 (1–4)	<0.01	3 (2–4)	3 (2–4)	3 (2–4)	3 (2–4)	0.08

Cut-points: men (Q1: <252; Q2: 252–306; Q3: 306–365; Q4: ≥365) and women (Q1: <203; Q2: 203–253; Q3: 253–304.7; Q4: ≥304.7).

SBP: systolic blood pressure; DBP: diastolic blood pressure; BMI: body mass index; TG: triglycerides; TC: total cholesterol; LDL-C: low density lipoprotein cholesterol; HDL-C: high density lipoprotein cholesterol; GLU, glucose; WBC, white blood cell; CHD, coronary heart disease; NIHSS, National Institute of Health Stroke Scale; mRS, modified Rankin Scale.

^*^Continuous variables are expressed as mean ± standard deviation, or as median (interquartile range). Categorical variables are expressed as frequency (percent).

^†^Baseline mRS score was determined at the time of enrollment.

**Table 2:** Odds ratios and 95% confidence intervals for primary outcome according to uric acid quartiles by sex among acute ischemic stroke patients.

	Uric acid, μmol/L				
	Q1	Q2	Q3	Q4	P_trend_
**Men**					
Primary outcome: death or major disability (mRS 3–6)					
No. of cases (%)	152 (29.1)	123 (23.6)	111 (21.3)	98 (18.7)	
Model 1	1.00	0.79 (0.59–1.05)	0.71 (0.53–0.95)	0.58 (0.43–0.79)	<0.01
Model 2	1.00	0.93 (0.67–1.30)	0.85 (0.60–1.20)	0.63 (0.44–0.91)	0.01
Secondary outcomes					
Major disability (mRS 3–5)					
No. of cases (%)	134 (25.7)	114 (21.9)	95 (18.2)	77 (14.7)	
Model 1	1.00	0.85 (0.63–1.13)	0.69 (0.51–0.94)	0.52 (0.38–0.72)	<0.01
Model 2	1.00	0.98 (0.72–1.35)	0.78 (0.56–1.09)	0.55 (0.38–0.78)	<0.01
Death					
No. of cases (%)	18 (3.5)	9 (1.7)	16 (3.1)	21 (4.0)	
Model 1	1.00	0.48 (0.21–1.11)	0.92 (0.45–1.89)	1.17 (0.59–2.32)	0.38
Model 2	1.00	0.58 (0.23–1.45)	1.08 (0.48–2.42)	1.33 (0.62–2.82)	0.30
**Women**					
Primary outcome: death or major disability (mRS 3–6)					
No. of cases (%)	83 (27.9)	71 (23.8)	82 (27.3)	87 (29.1)	
Model 1	1.00	0.84 (0.57–1.24)	0.95 (0.65–1.39)	0.97 (0.66–1.42)	0.98
Model 2	1.00	1.05 (0.67–1.64)	1.40 (0.90–2.18)	1.29 (0.83–2.01)	0.15
Secondary outcomes					
Major disability (mRS 3–5)					
No. of cases (%)	73 (24.5)	65 (21.8)	70 (23.3)	78 (26.1)	
Model 1	1.00	0.89 (0.60–1.33)	0.92 (0.62–1.37)	1.02 (0.69–1.50)	0.90
Model 2	1.00	1.05 (0.68–1.62)	1.20 (0.78–1.85)	1.26 (0.82–1.93)	0.23
Death					
No. of cases (%)	10 (3.4)	6 (2.0)	12 (4.0)	9 (3.0)	
Model 1	1.00	0.68 (0.24–1.94)	1.22 (0.50–2.99)	0.86 (0.33–2.25)	0.95
Model 2	1.00	0.78 (0.26–2.34)	1.68 (0.64–4.43)	1.14 (0.41–3.17)	0.52

Cut-points: men (Q1: <252; Q2: 252–306; Q3: 306–365; Q4: ≥365) and women (Q1: <203; Q2: 203–253; Q3: 253–304.7; Q4: ≥304.7).

Model 1, adjusted for age, time from onset to hospitalization, current smoking, alcohol consumption, glucose, SBP, WBC, dyslipidemia, history of hypertension, history of coronary heart disease, history of diabetes mellitus, family history of stroke, and current use of antihypertensive medications; Model 2, further adjusted for baseline NIHSS score.

**Table 3:** Multivariate ORs (95% CIs) of clinical outcomes associated with each SD increase of serum uric acid in both men and women*.

	Model 1		Model 2	
	OR (95% CI)	*P* value	OR (95% CI)	*P* value
Men				
Death or major disability (mRS 3–6)	0.83 (0.75–0.93)	0.001	0.86 (0.76–0.98)	0.020
Major disability (mRS 3–5)	0.80 (0.72–0.90)	<0.001	0.83 (0.73–0.93)	0.002
Modified Rankin Scale^a^	0.87 (0.80–0.94)	<0.001	0.94 (0.87–1.02)	0.130
Death	1.08 (0.83–1.41)	0.571	1.17 (0.90–1.52)	0.236
Women				
Death or major disability (mRS 3–6)	0.96 (0.84–1.10)	0.576	1.05 (0.90–1.23)	0.540
Major disability (mRS 3–5)	0.94 (0.82–1.09)	0.418	1.00 (0.86–1.16)	0.996
Modified Rankin Scale^a^	0.90 (0.81–1.00)	0.055	0.94 (0.85–1.05)	0.276
Death	1.16 (0.85–1.60)	0.352	1.25 (0.89–1.75)	0.192

Model 1, adjusted for age, time from onset to hospitalization, current smoking, alcohol consumption, glucose, SBP, WBC, dyslipidemia, history of hypertension, history of coronary heart disease, history of diabetes mellitus, family history of stroke, and current use of antihypertensive medications; Model 2, further adjusted for baseline NIHSS score.

^*^Men: SD = 96.1 μmol/L; women: SD = 89.9 μmol/L.

^a^Odds of a 1-unit higher modified Rankin score.

**Table 4:** Subgroup analysis of adjusted ORs (95% CIs) of primary outcome according to uric acid quartiles.

Subgroup	Uric acid (μmol/L)					
	Q1	Q2	Q3	Q4	P_trend_	P_interaction_
Men	<252	252–306	306–365	≥365		
Stroke onset age, years						0.06
<60	1.00	1.06 (0.59–1.92)	0.61 (0.33–1.15)	0.44 (0.22–0.86)	0.01	
≥60	1.00	0.80 (0.53–1.21)	0.95 (0.62–1.44)	0.71 (0.46–1.10)	0.22	
Baseline SBP, mm Hg						0.21
<160	1.00	0.78 (0.45–1.36)	0.61 (0.35–1.07)	0.54 (0.30–0.97)	0.02	
≥160	1.00	1.04 (0.68–1.59)	1.08 (0.69–1.69)	0.70 (0.44–1.12)	0.20	
Cigarette smoking						0.42
No	1.00	0.84 (0.52–1.35)	0.89 (0.54–1.46)	0.68 (0.41–1.13)	0.18	
Yes	1.00	1.01 (0.63–1.62)	0.77 (0.47–1.27)	0.56 (0.33–0.94)	0.02	
Alcohol consumption						0.46
No	1.00	0.67 (0.44–1.03)	0.83 (0.54–1.27)	0.68 (0.44–1.05)	0.14	
Yes	1.00	1.13 (0.69–1.84)	0.84 (0.51–1.39)	0.57 (0.34–0.97)	0.02	
Receiving immediate BP reduction						0.18
No	1.00	1.07 (0.70–1.66)	0.79 (0.51–1.24)	0.55 (0.34–0.88)	0.01	
Yes	1.00	0.69 (0.44–1.10)	0.83 (0.52–1.33)	0.74 (0.46–1.18)	0.29	
Women	<203	203–253	253–304.7	≥304.7		
Stroke onset age, years						0.90
<60	1.00	1.08 (0.53–2.20)	2.00 (1.01–3.97)	0.96 (0.44–2.09)	0.53	
≥60	1.00	0.94 (0.51–1.72)	1.01 (0.55–1.84)	1.42 (0.81–2.49)	0.19	
Baseline SBP, mm Hg						0.64
<160	1.00	3.30 (1.29–8.45)	3.70 (1.46–9.42)	2.09 (0.84–5.24)	0.16	
≥160	1.00	0.84 (0.44–1.27)	1.04 (0.62–1.74)	1.12 (0.67–1.89)	0.45	
Cigarette smoking						0.13
No	1.00	1.01 (0.64–1.61)	1.23 (0.78–1.96)	1.21 (0.76–1.91)	0.32	
Yes	1.00	4.68 (0.39–55.44)	13.41 (1.27–141.49)	6.87 (0.64–73.40)	0.10	
Alcohol consumption						0.26
No	1.00	1.05 (0.67–1.66)	1.40 (0.89–2.20)	1.36 (0.87–2.13)	0.10	
Yes	1.00	—	—	—	NA	
Receiving immediate BP reduction						0.86
No	1.00	1.15 (0.60–2.19)	1.73 (0.92–3.25)	1.39 (0.73–2.65)	0.19	
Yes	1.00	1.03 (0.54–1.98)	1.24 (0.65–2.37)	1.35 (0.72–2.53)	0.29	

NA: not available for limited sample size.

In the multivariate models, confounding factors such as age, time from onset to hospitalization, current smoking, alcohol consumption, glucose, SBP, WBC, dyslipidemia, history of hypertension, history of coronary heart disease, history of diabetes mellitus, family history of stroke, current use of antihypertensive medications, baseline NIHSS score and randomized treatment were included unless the variable was used as a subgroup variable.
