# Supplementary material for: Sex-specific Association Between Uric Acid and Outcomes After Acute Ischemic Stroke: A Prospective Study from CATIS Trial
Source: Sci Rep. 2016 Nov 30;6:38351. doi: 10.1038/srep38351 (PMC5128785; doi:10.1038/srep38351)
Supplement: Supplementary Tables [file srep38351-s1.pdf]

Sex-specific Association Between Uric Acid and Outcomes After Acute Ischemic Stroke: A Prospective Study from CATIS Trial

Li-Hua Chen, PhD[1,#]; Chongke Zhong, MD[1,2,#]; Tan Xu, MD, PhD[1,2]; Tian Xu, MD, PhD[1,3]; Yanbo Peng, MD, PhD [4]; Aili Wang MD, PhD[1]; Jinchao Wang, MD[5]; Hao Peng, MD, PhD [1]; Qunwei Li, MD, PhD [6]; Zhong Ju, MD, PhD[7]; Deqin Geng, MD[8]; Jintao Zhang, MD, PhD [9]; Yongqiu Li, MD, PhD [10]; Yonghong Zhang, MD, PhD[1,2,*]; Jiang He, MD, PhD[1,2,*].

1. Department of Epidemiology, School of Public Health and Jiangsu Key Laboratory of Preventive and Translational Medicine for Geriatric Diseases, Medical College of Soochow University, Suzhou, China.

2. Department of Epidemiology, Tulane University School of Public Health and Tropical Medicine, New Orleans, LA.

3. Department of Neurology, Affiliated Hospital of Nantong University, Nantong, Jiangsu, China.

4. Department of Neurology, Affiliated Hospital of North China University of Science and Technology, Hebei, China.

5. Department of Neurology, Yutian County Hospital, Hebei, China.

6. Department of Epidemiology, School of Public Health, Taishan Medical College, Shandong, China.

7. Department of Neurology, Kerqin District First People’s Hospital of Tongliao City, Inner Mongolia, China

8. Department of Neurology, Affiliated Hospital of Xuzhou Medical College, Jiangsu,

China.

9. Department of Neurology, the 88th Hospital of PLA, Shandong, China

10. Department of Neurology, Tangshan Worker's Hospital, Hebei, China

[#] **These authors contributed equally to this work.**

***Correspondence to:**

Yonghong Zhang, MD, PhD, Department of Epidemiology, School of Public Health and Jiangsu Key Laboratory of Preventive and Translational Medicine for Geriatric Diseases, Medical College of Soochow University, 199 Renai Road, Industrial Park District, Suzhou, Jiangsu Province 215123, China. Tel: +86 512 6588 0078; E-mail: yhzhang@suda.edu.cn

Or

Jiang He, MD, PhD, Department of Epidemiology, Tulane University School of Public Health and Tropical Medicine, New Orleans, USA. (504) 988-5165;

E-mail: jhe@tulane.edu

Supplemental Table I. Baseline characteristics of study participants.

Characteristics	Male	Female	P value	All
Number of subjects	2089	1195		3284
Age, mean ± SD, y	61.3 ± 11.1	64.6 ± 10.6	<0.001	62.5 ± 11.0
Time from onset to randomization, mean ± SD, h	15.1 ± 13.1	15.6 ± 13.2	0.302	15.3 ± 13.1
SBP, mean ± SD, mm Hg	165.7 ± 16.8	167.2 ± 17.1	0.014	166.2 ± 17.0
DBP, mean ± SD, mm Hg	97.4 ± 11.3	95.2 ± 10.7	<0.001	96.6 ± 11.1
BMI, mean ± SD, kg/m^2	24.9 ± 2.7	25.2 ± 3.3	0.014	25.0 ± 3.0
TG, median (IQR), mmol/L	1.4 (1.0-2.1)	1.6 (1.1-2.2)	<0.001	1.5 (1.0-2.1)
TC, median (IQR), mmol/L	4.8 (4.2-5.5)	5.3 (4.6-6.1)	<0.001	5.0 (4.3-5.7)
LDL-C, median (IQR), mmol/L	2.8 (2.2-3.4)	3.1 (2.5-3.7)	<0.001	2.9 (2.3-3.5)
HDL-C, median (IQR), mmol/L	1.2 (1.0-1.4)	1.3 (1.1-1.5)	<0.001	1.2 (1.0-1.5)
GLU, median (IQR), mmol/L	5.7 (5.0-7.0)	5.9 (5.1-7.7)	<0.001	5.8 (5.1-7.2)
History of hypertension, No (%)	1635 (78.3)	977 (81.8)	0.017	2612 (79.5)
Current use of antihypertensive medications, No (%)	974 (46.6)	660 (55.2)	<0.001	1634 (49.8)
History of hyperlipidemia, No (%)	135 (6.5)	87 (7.3)	0.369	222 (6.8)
History of diabetes mellitus, No (%)	351 (16.8)	243 (20.3)	0.011	594 (18.1)
History of coronary heart disease, No (%)	209 (10.0)	172 (14.4)	<0.001	381 (11.6)
History of chronic kidney disease, No (%)	21 (1.0)	13 (1.1)	0.822	34 (1.0)
Family history of stroke, No (%)	416 (19.9)	240 (20.1)	0.907	656 (20.0)
Current cigarette smoking, No (%)	1137 (54.4)	117 (9.8)	<0.001	1254 (38.2)
Current alcohol drinking, No (%)	988 (47.3)	37 (3.1)	<0.001	1025 (31.2)
Antihypertensive therapy, No (%)	1056 (50.6)	595 (49.8)	0.675	1651 (50.3)
Ischemic stroke subtype, No (%)				
Thrombotic	1655 (79.2)	938 (78.5)	0.621	2593 (79.0)
Embolic	60 (2.9)	42 (3.5)	0.307	102 (3.1)
Lacunar	421 (20.2)	252 (21.1)	0.523	673 (20.5)
Baseline NIHSS score, median (IQR)	4 (2-7)	4 (3-8)	0.064	4 (2-7)
Baseline mRS score, median (IQR)	3 (2-4)	3 (2-4)	0.010	3 (2-4)
Creatinine, mean ± SD, μmol/L	77.3 ± 25.4	61.7 ± 24.5	<0.001	71.6 ± 26.1
WBC, mean ± SD, 10^9/L	8.1 ± 26.6	7.8 ± 21.1	0.704	8.0 ± 24.7
Uric acid, mean ± SD, μmol/L	310.6 ± 96.1	257.5 ± 89.9	<0.001	291.3 ± 97.3

SBP: systolic blood pressure; DBP: diastolic blood pressure; BMI: body mass index; TG: triglyceride; TC: total cholesterol; LDL-C: low density lipoprotein cholesterol; HDL-C: high density lipoprotein cholesterol; GLU, glucose; NIHSS, National Institute of Health Stroke Scale; mRS, modified Rankin Scale; WBC, white blood cell.

Supplemental Table II. Multiple-adjusted odds ratio (ORs) and 95% confidence intervals (CIs) of primary outcome according to quartile of serum uric acids: sensitivity analyses.

	Uric acid (μmol/L)					
Variable	Q1	Q2	Q3	Q4	P_{trend}	$P_{interaction}$*
Men	<252	252-306	306-365	≥365		
Model 1	1.00	0.93(0.67-1.30)	0.85(0.60-1.20)	0.63(0.44-0.91)	0.01	0.0069
Model 3	1.00	0.93(0.67-1.30)	0.85(0.60-1.20)	0.63(0.44-0.91)	0.01	0.0073
Model 2	1.00	0.95(0.68-1.33)	0.87(0.61-1.23)	0.65(0.44-0.94)	0.02	0.0069
Model 4	1.00	0.89(0.64-1.24)	0.83(0.59-1.18)	0.63(0.44-0.90)	0.01	0.0054
Women	<203	203-253	253-304.7	≥304.7		
Model 1	1.00	1.05(0.67-1.64)	1.40(0.90-2.18)	1.29(0.83-2.01)	0.15	
Model 3	1.00	1.05(0.67-1.64)	1.40(0.90-2.18)	1.29(0.83-2.01)	0.15	
Model 2	1.00	1.07(0.68-1.69)	1.44(0.92-2.25)	1.34(0.85-2.11)	0.11	
Model 4	1.00	1.05(0.67-1.66)	1.39(0.89-2.17)	1.35(0.86-2.11)	0.11	

Model 1: adjusted for age, time from onset to hospitalization, current smoking, alcohol consumption, glucose, SBP, WBC, dyslipidemia, history of hypertension, history of coronary heart disease, history of diabetes mellitus, family history of stroke, current use of antihypertensive medications, and baseline NIHSS score.

Model 2: adjusted for Model 1 and further adjusted for randomized treatment.

Model 3: adjusted for Model 1 and further adjusted for baseline serum creatinine.

Model 4: adjusted for Model 1 and further excluded individuals with chronic kidney disease.

* P value for interaction between serum UA and sex.
